# Reintroduction and Post-Release Survival of a Living Fossil: The Chinese Giant Salamander

**DOI:** 10.1371/journal.pone.0156715

**Published:** 2016-06-03

**Authors:** Lu Zhang, Wei Jiang, Qi-Jun Wang, Hu Zhao, Hong-Xing Zhang, Ruth M. Marcec, Scott T. Willard, Andrew J. Kouba

**Affiliations:** 1 Biochemistry, Molecular Biology, Entomology, and Plant Pathology Department, Mississippi State University, Mississippi State, Mississippi, United States of America; 2 Conservation and Research Department, Memphis Zoological Society, Memphis, Tennessee, United States of America; 3 Shaanxi Institute of Zoology, Xi’an, Shaanxi, China; 4 Department of Wildlife, Fisheries, and Aquaculture, Mississippi State University, Mississippi State, Mississippi, United States of America; CNRS, FRANCE

## Abstract

Captive rearing and reintroduction / translocation are increasingly used as tools to supplement wild populations of threatened species. Reintroducing captive-reared Chinese giant salamanders may help to augment the declining wild populations and conserve this critically endangered amphibian. We released 31 captive-reared juvenile giant salamanders implanted with VHF radio transmitters at the Heihe River (n = 15) and the Donghe River (n = 16) in the Qinling Mountains of central China. Salamanders were monitored every day for survival from April 28^th^ 2013 to September 3^rd^ 2014. We attempted to recapture all living individuals by the end of the study, measured their body mass and total body length, and checked for abnormalities and presence of external parasites. Two salamanders at the Heihe River and 10 animals at the Donghe River survived through the project timeline. Nine salamanders were confirmed dead, while the status of the other 10 animals was undetermined. The annual survival rate of giant salamanders at the Donghe River (0.702) was 1.7-fold higher than that at the Heihe River (0.405). Survival increased as individuals were held longer following surgery, whereas body mass did not have a significant impact on survival rate. All salamanders recaptured from the Donghe River (n = 8) increased in mass (0.50 ± 0.13 kg) and length (5.5 ± 1.5 cm) after approximately 11 months in the wild, and they were only 7% lighter than wild animals of the same length (mean residual = -0.033 ± 0.025). Our results indicate that captive-reared Chinese giant salamanders can survive in the wild one year after release and adequate surgical recovery time is extremely important to post-release survival. Future projects may reintroduce older juveniles to achieve better survival and longer monitoring duration.

## Introduction

Amphibians have been facing widespread population declines since the 1970s with over 32% of amphibian species currently threatened worldwide [1]. These population declines are due to habitat loss, pollution, over-consumption and disease (both fungal and viral), which have been further impacted by climate change [2,3]. To counter balance these declines, captive rearing and repatriation are increasingly used as tools to supplement wild populations of threatened amphibians [4]. However, reintroduction and translocation programs for amphibians have had very low success, especially when viewed through the lens of a self-sustaining and reproducing wild population [5,6]. The success of a reintroduction project is reported to be related to the number of animals released, with projects releasing over 1000 individuals being the most successful [6]. Thus, a sustainable source population with numerous individuals in captivity must be established before any reintroduction project becomes feasible.

China has a rich diversity of endemic amphibian species, and similar to global trends, many populations are declining in the wild. These declines are at a slightly lower level than global rates; 27% of amphibians in China are threatened [7]. Among the most endangered species, the Chinese giant salamander (*Andrias davidianus*) is the world’s largest amphibian [8], reaching up to 1.8 meters in length and weighing up to 50 kg [9,10]. Often referred to as a living fossil, it is one of three species belonging to the family Cryptobranchidae, which have diverged from all other salamander lineages in the middle Jurassic [11] or early Cretaceous [12] periods. The species was once found in vast areas of central and southern China, living in various water bodies which included streams, rivers, and underground rivers in karst caves [9]. The species has suffered an 80% population decline since the 1950s on account of habitat destruction, water pollution, and over-exploitation for its flesh [13], and was listed as critically endangered on the IUCN Red List in 2004. Over the last decade, a farming industry for giant salamanders has rapidly developed due to the high market price of its meat. These farms are supported by county and provincial governments as economic enterprises to help generate income and support local villages. Although not reported in scientific journals, some *Andrias* farms have gained significant experience rearing and reproducing these salamanders (e.g. one farm is producing over 20,000 larvae per year). Thus, *Andrias* farms could provide a large and stable source population for reintroduction programs throughout the country if managed correctly. Hence, the Chinese giant salamander provides a unique opportunity to reintroduce captive-bred individuals to augment and restore declining wild populations.

Over the last decade, a small number of giant salamander reintroduction projects have taken place in China, which was either launched by nature reserves or regional governmental agencies (e.g. [14]). However, there has been little monitoring work on the salamanders’ survival in the wild. One study monitored survival of four adult giant salamanders released in a stream pool in Lushi County, Henan Province [15]. These animals were monitored for 5 months using external VHF radio transmitters and subsequently retrieved and returned to captivity upon completion of the study. To our knowledge, no free movement studies, utilizing internal VHF radio transmitters, where the animals were left in the stream as part of the native population have been conducted.

The aim of our project was to test whether captive-reared Chinese giant salamanders were suitable for reintroduction, and determine how well they survived in the wild post-release. For this study, we selected two rivers where giant salamanders were historically observed, with the assumption that these rivers could still support salamander populations. By tracking the salamanders using implanted VHF radio transmitters, we were able to monitor them for approximately one year in the wild, record their survival over time, and compare body condition pre- and post-release. Moreover, we were able to evaluate factors that may be associated with mortality of the animals in the wild, as well as model determinant factors that may impact the success of the reintroduction. The findings from this study will provide valuable criteria for similar reintroduction projects in the future that are meant to aid in the restoration of wild Chinese giant salamander populations.

## Materials and Methods

We used captive reared juvenile giant salamanders as study animals, with appropriate ethics and protocols approved by the Shaanxi Province Department of Water Resources and the Shaanxi Institute of Zoology. As the lead institute for this project in China, the Shaanxi Institute of Zoology animal research committee reviewed the proposed work and agreed that the study met all animal welfare requirements for Shaanxi Province related to Fisheries and Aquaculture. The Fisheries Bureau of Shaanxi Province, under the Department of Water Resources, provided a permit to our team for reintroduction of the Chinese giant salamanders at both study sites, after reviewing the protocol.

### Study Area

**Fig 1:**
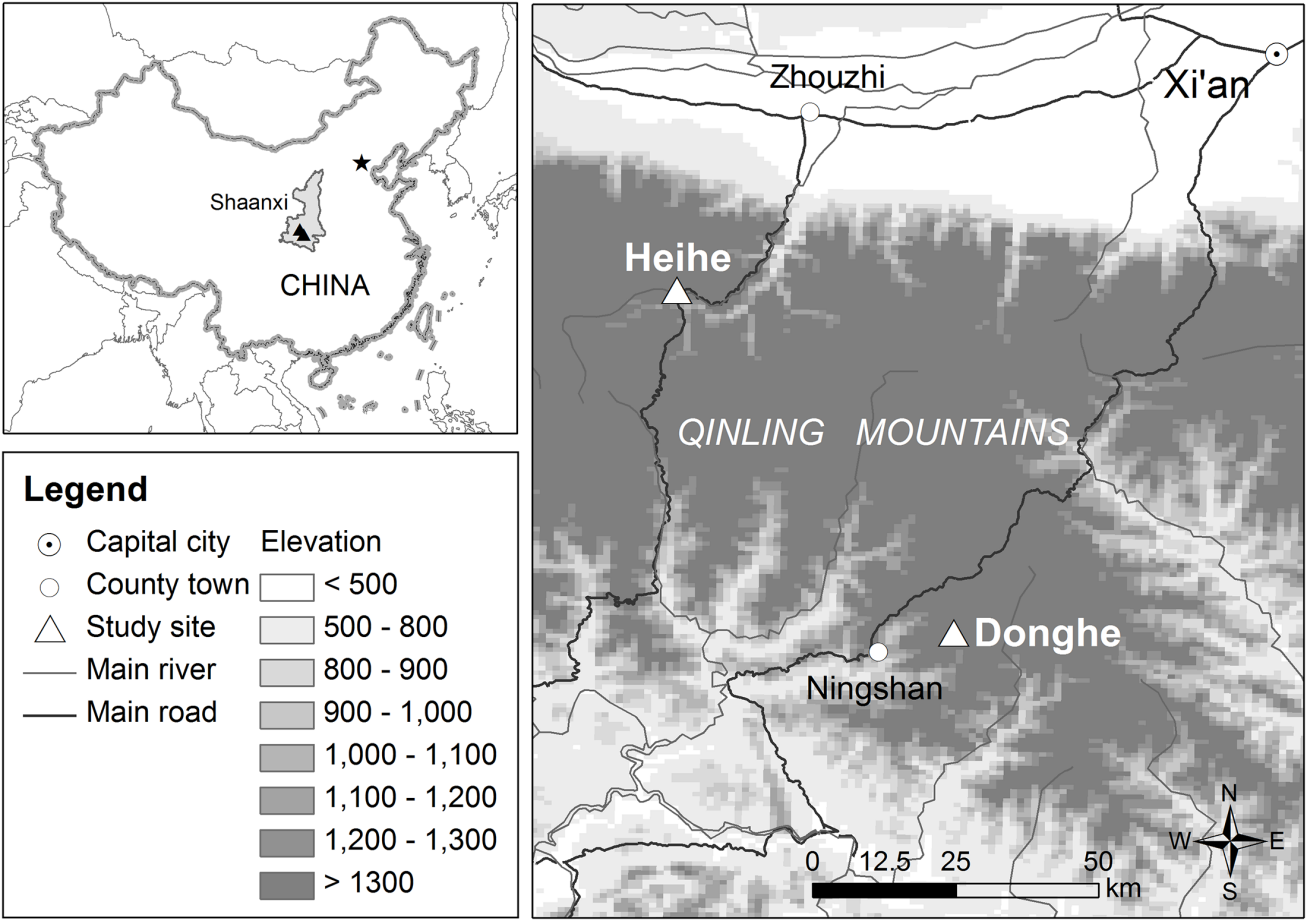
General location of the study sites. We reintroduced 31 juvenile Chinese giant salamanders in two rivers within the Qinling Mountains in central China. Details of reintroduction locations are not displayed so as to protect released animals from possible disturbance or poaching.

The two rivers selected for this study were the Heihe River and Donghe River, in the Qinling Mountains, Shaanxi Province, China (Fig 1). The Heihe River, on the north slope of the mountains, belongs to the Weihe River watershed, which is the largest branch of the Yellow River. The vegetation along the river is mainly deciduous broad-leaf forest. The Donghe River, on the south slope of the Mountains, belongs to the Hanjiang River watershed, which is the largest branch of the Yangtze River. The vegetation along the Donghe River is a mixture of ever-green and deciduous broad-leaf forest. Both rivers provided natural habitat and robust populations of giant salamanders in the past. Giant salamander larvae are still collected every year from the exit of an underground river section at Heihe, close to the site where we released our animals. No giant salamanders have been found in the Donghe River for years, although one sub-adult animal was found close to our release site during the study, suggesting there may still be wild giant salamanders in the river, but at a very low density.

### Study Animals and Transmitter Implantation

Thirty-two juvenile giant salamanders were purchased from two farms—Longquan *Andrias* Farm (Ningshan) and Kangxingyan *Andrias* Farm (Xi’an)–within the Qinling Mountains for this study. Animals were selected based on the fact that they originated from one of the two sites where we planned to reintroduce the animals. The 16 salamanders for release at the Heihe River were collected as larvae at the exit of the underground river by the farm in 2010 and reared in captivity thereafter. Their body mass ranged from 0.36 to 1.14 kg at the time of surgical implantation of VHF radio transmitters (F1170, Advanced Telemetry Systems Inc., Isanti, MN, USA). The 16 salamanders for release at the Donghe River were born in captivity in 2008, and their parents were originally collected from this river. Their body mass ranged from 1.10 to 2.34 kg at time of surgical implantation. VHF radio transmitters were surgically implanted into the coelomic cavity of all 32 animals. Each animal had a unique radio frequency (150.1–150.8 MHz) allowing for individual identification. Transmitters measured approximately 24×14×7 mm, and weighed 4 g, which was well below the limitation of ≤ 5% of the salamander body mass [16]. The battery life of the transmitters was estimated at 14 months by the company. In addition, released salamanders were marked with Passive Integrated Transponder (PIT) tags (Biomark Inc., Boise, ID, USA) allowing for an additional level of identification following recapture, especially past the lifespan of the transmitters. VHF radio transmitter implantation was conducted by a professional veterinarian between March 13–16, 2013 and details for the surgery can be found in Marcec *et al*. [17].

### Biometric Data Collection and Post-Release Monitoring

We recorded biometric data on the 32 salamanders at time of surgery and before release, including body mass (kg), snout-vent length (SVL) and total body length (TBL) in cm, any abnormalities and presence of external parasites. Prior to release, two of the 16 giant salamanders prepared for the Heihe River experienced dehiscence of the suture site. These animals had their surgical incision re-sutured and they were held back from release to heal. One of the animals died three weeks after dehiscence, while the other was released on November 5^th^, 2013, six months after the initial animals were discharged. Therefore, a total of 14 animals were initially released at the Heihe River April 28 –May 2, 2013. The remaining 16 animals were released at the Donghe River on July 12, 2013. With the help of local field assistants, we located released animals every day by tracking them using a three element Yagi antenna and a handheld receiver (R410, Advanced Telemetry Systems, Isanti, MN, USA). The coordinates of individual salamanders were collected by global positioning system (GPS) devices (GPS 60CSx, Garmin Corp., New Taipei City, Taiwan). Individuals that continued to move actively were considered alive by the last time movement was recorded. Observations were also taken by an underwater inspection camera (M12, Milwaukee Electric Tool, Brookfield, WI, USA) to confirm presence of the salamander and its status. Deaths were not confirmed unless identified by visual observation.

Near the end of the study, recapture of all living individuals was attempted before the radio signal disappeared, using pot-fishing nets with chopped chicken breast as bait. We did not apply the traditional bow-hooks method to catch the giant salamanders, as this device has been known to cause severe injury. A total of 4 traps were set at the Heihe River, whereas 14 traps were set at the Donghe River to capture giant salamanders. Salamander traps were set in the evening beside rocks where giant salamanders had been located via telemetry, and checked the next morning. When a giant salamander was recaptured, we recorded its body mass, SVL, TBL, any abnormalities, and external parasites so as to compare to pre-release data. All recaptured giant salamanders were released at the same location where they were caught once all measurements were completed.

Prey species and abundance at both rivers were surveyed in June 2014 using similar trapping mechanisms as those used for the salamanders. Traps were set in the evening, 50 m apart from adjacent traps and checked the next morning. Based on the length of river section that salamanders occupied, a total of 14 traps were set at the Heihe River, whereas 30 traps were set at the Donghe River. We recorded prey species collected, number of individuals, and mass of all animals captured in the traps, and then released them at the same location where they were caught.

### Data Analysis

We used the known-fate model, with a logit link function in Program MARK [18], to estimate survival rates of reintroduced giant salamanders. The main monitoring period (April 28, 2013 –June 30, 2014) was divided into 14 intervals (each interval corresponds to a month) for which survival probability could be estimated. Radio signals gradually disappeared beginning June, 2014, and the last signal was collected on September 3^rd^, 2014. We left-censored individuals until the day they were released (to exclude animals that had died prior to release, thus survival calculations were started following the release), and right-censored data if the animal’s radio signal disappeared and fate was undetermined (n = 10). We considered five variables that may affect the survival of released giant salamanders: two group variables including site (Heihe River vs. Donghe River) and age (3-year-old vs. 5-year-old), one time covariate which was months post-release, and two individual covariates including initial body mass, and days held from surgery to release (DSR). We first compared the site model and the age model and found the two had equal support. Thus, we retained site for generating candidate models as our main group variable. Each model represented monthly survival rate of the salamanders as a function of some combination of variables. We used Akaike’s Information Criterion for small sample sizes (AICc; [19]) to assess the relative support among candidate models, and considered models with ≤ 2 delta AICc as having the same level of best support. We calculated Akaike weight (ω_*i*_) for each candidate model and applied a model-averaging approach if no single model was superior to the others (i.e., ω_max_ > 0.9).

All giant salamanders recaptured at the end of the study were compared for differences in body mass, SVL, and TBL to their original values prior to release. We applied non-parametric Wilcoxon’s signed ranks test to compare paired data after testing for normality and homogeneity of variances using the Shapiro-Wilk and Bartlett’s tests, respectively.

The 16 giant salamanders released in the Donghe River were from a group of siblings (n = 240) that were monitored beginning in 2009 when these animals were in their second year of life, with the help of PIT tags for individual identification. Their biometric data were recorded at six intervals between 2009 and 2013 [20]. In June 2014, we randomly selected 34 individuals from the group that remained in captivity and recorded their biometric data. Reintroduced salamanders were compared with these 34 animals in body mass and TBL at two intervals—before they were separated for this project in November 2012 and at the end of the study in June 2014.

We also compared body condition of the 32 giant salamanders for reintroduction with wild caught animals as reported in the literature: body mass and TBL of reintroduced salamanders were plotted against the regression line constructed using previously published data (body mass and TBL) collected on wild caught giant salamanders [20], and their residuals were calculated. Individuals with positive residual scores were considered to be in better body condition than wild animals, whereas individuals with negative residual scores were considered to be in worse body condition [16,21,22]. For animals at the Heihe River, we only compared their residuals at surgery (March 15, 2013) and pre-release (April 28, 2013) since no animals were recaptured from this location. For animals at the Donghe River, we compared their residuals at surgery (March 15, 2013), pre-release (July 7, 2013) and at recapture (June 12, 2014). We then converted residuals of recaptured animals to body mass differences between them and wild specimens having the same TBL using the following equation: 10^residual^ = Mass_captive_ / Mass_wild_ [20].

Mass of prey species per trap was compared between the Heihe and Donghe rivers. We applied a Mann-Whitney U test to compare differences between two groups and a Kruskal-Wallis test to compare differences among three groups. All tests for normality, homogeneity and statistical comparisons were conducted in SPSS 22.0.

## Results

### Survival

Out of the 15 giant salamanders released at the Heihe River, six died, seven were status undetermined, while only two were confirmed alive by visual observation using the underwater camera, and could be tracked normally until the end of our project (S1 Table). Among the six dead salamanders, four experienced dehiscence of the suture site and were recaptured, treated for their injury, but subsequently died from the trauma. The fifth salamander died after a flood with broken limbs and viscera, while the cause of death for the sixth salamander was indeterminable; however, the effect of a freshwater fungus *Saprolengnia* may have been involved. All mortalities (n = 6) occurred within 50 days post-release. Salamanders with undetermined status included five animals that were washed downstream by floods and their signals disappeared within our search radius (20 km downstream), one animal whose radio signal disappeared within one day without any floods having occurred, and one animal that moved into an underground stream where we lost its signal.

Giant salamanders at the Donghe River had a better survivorship; here only three animals were confirmed dead, three were status undetermined, and 10 were alive and traceable by the end of the study (S1 Table). Bodies of two dead salamanders were retrieved from the river; however, the cause of death could not be determined upon necropsy. The third dead animal was never retrieved and was last observed in November 2013 with severe external wounds and a skinny body. We considered it dead in December 2013 as its location had not changed for a month and we eventually found its radio signal coming from a dry river bank when the water was shallow during the winter. All mortalities (n = 3) occurred between 90 to 180 days post-release. Salamanders with undetermined status included two animals that were washed downstream by floods, and one animal whose radio signal disappeared within one day without any floods having occurred. The two animals washed downstream by the flood were found moving for several months following the event; however, they were never recaptured, nor re-sighted by the end of the study, thus we considered them as status undetermined by the end of the study. Among the 10 live salamanders, eight were recaptured in traps while another two were confirmed alive by visual observation using the underwater camera by the end of the study. One of the trapped animals was also washed downstream by floods; however, it remained traceable for several months before we recaptured it near the end of the study.

**Fig 2:**
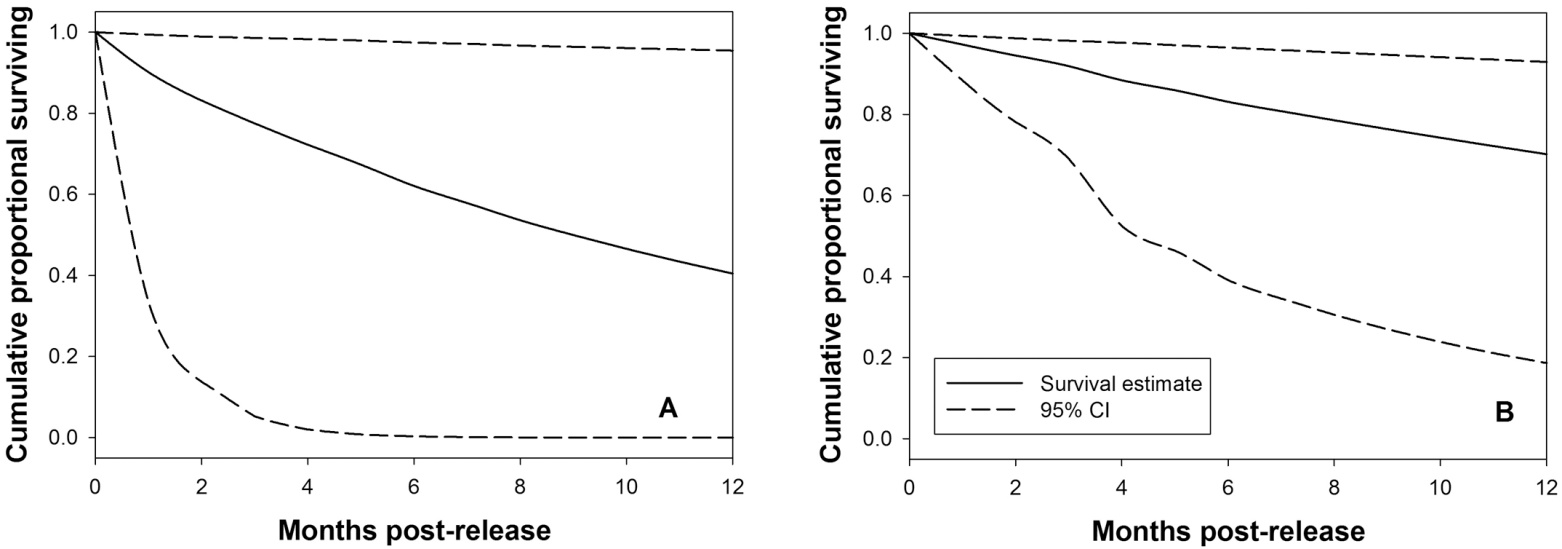
Survival rate estimates from MARK of reintroduced salamanders over one year. Salamanders reintroduced to the Heihe River (A) had a lower annual survival estimate than salamanders reintroduced to the Donghe River (B).

**Fig 3:**
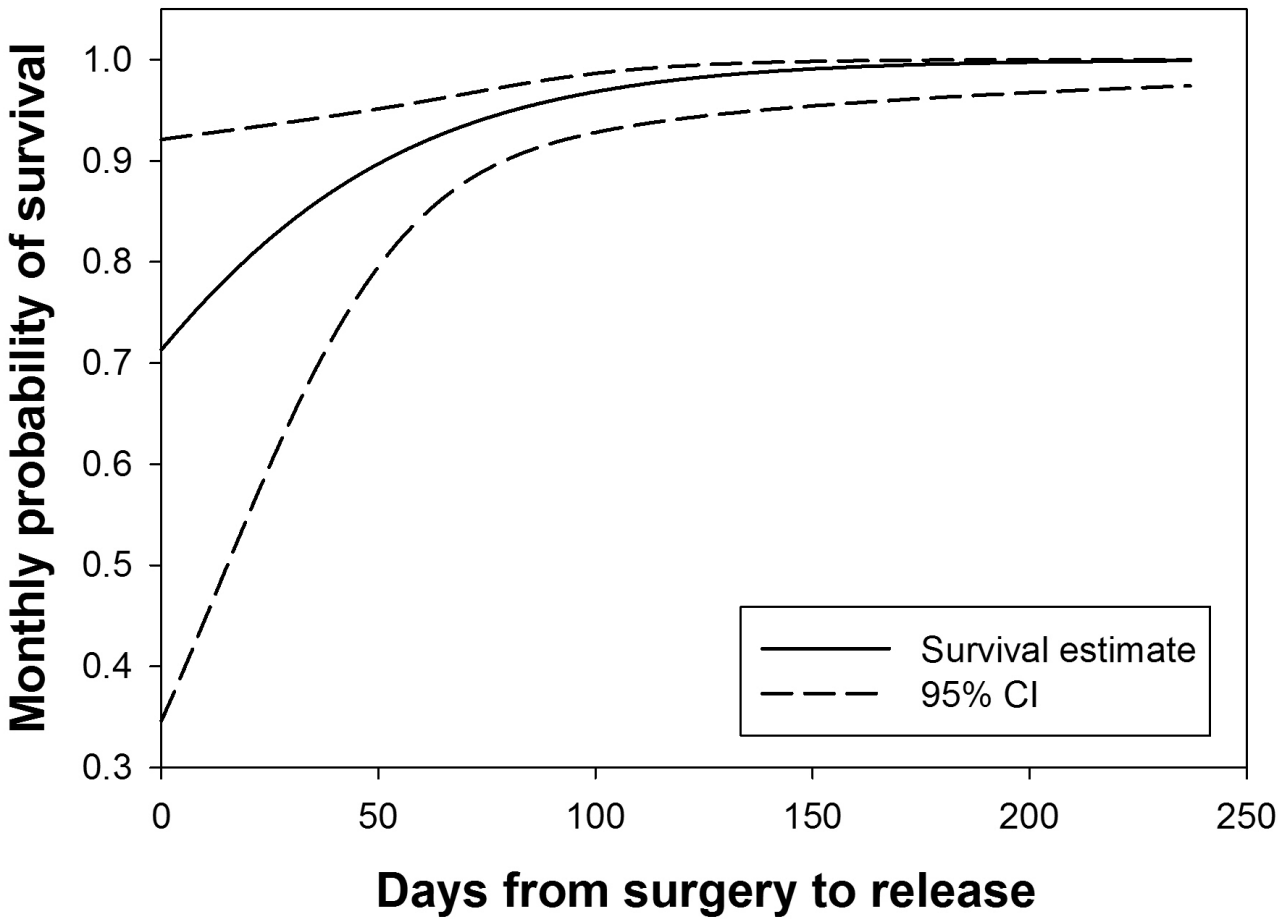
Survival probability of reintroduced salamanders increased as they were held longer from surgery to release. Combining the two groups of salamanders, analysis in MARK indicated that the number of days held from surgery to release was important to increase post-release survival of reintroduced salamanders.

Survival analyses in MARK indicated that the DSR model and the site model were the most supported (with ≤ 2 ΔAIC). However, because they had a cumulative Akaike weight of approximately 0.66 (Table 1), we applied a model-averaging procedure to derive monthly survival rate of the salamanders. Monthly survival rate varied slightly by month at both sites, with a higher average rate of 0.971 ± 0.001 SE at the Donghe River than 0.928 ± 0.002 SE at the Heihe River (*P* < 0.001). The annual survival rate of giant salamanders at the Donghe River (0.702) was about 1.7-fold higher than at the Heihe River (0.405, Fig 2). Besides site, DSR was included in the most supported models, indicating that survival increased as salamanders were held longer following surgery. For example, monthly survival rate of giant salamanders would be over 98% if they were held more than 120 days (Fig 3). Thus, adequate time for healing of the suture site to prevent dehiscence was extremely important. Body mass of the animals at time of release did not have an impact on their survival since it was not included in the best supported models.

**Table 1:** Model selection results for survival analysis of captive-reared Chinese giant salamanders reintroduced in two rivers in the Qinling Mountains, Shaanxi Province, China, 2013–2014.

Model	k	AICc	ΔAICc	*ω*_*i*_	Deviance
S(DSR)	2	71.180	0.000	0.444	67.122
S(site)	2	72.599	1.419	0.218	68.542
S(site × DSR)	4	74.231	3.051	0.097	66.039
S(mass)	2	74.392	3.213	0.089	70.335
S(month)	14	74.446	3.266	0.087	44.325
S(site × mass)	4	74.997	3.818	0.066	66.805
S(site × month)	28	108.185	37.006	0.000	43.359

k, Number of parameter; *ω*_*i*_, Akaike weight; DSR, days held from surgery to release; mass, body mass at time of release; month, months post-release.

### Battery Life and Transmission Range of Transmitters

Based on the 12 giant salamanders that were monitored through the end of our project at both rivers, we found the average battery life of the VHF radio transmitters was 482 days (range: 459–537 days). This was longer than the expected battery life, which was 441 days according to the product description from the company. We found the battery life to be exceptional for the size of the transmitters (weight = 4 g). Radio signals were best received within ~300 m, shorter than the anticipated range provided by the company, which was about 800 m. However, this transmission range was sufficient for monitoring aquatic species in rivers in mountainous areas.

### Growth and Body Condition

All of the recaptured animals at the Donghe River (n = 8) had grown longer and heavier after 11 months in the wild, with a mean body mass increase of 0.50 ± 0.13 kg (range: 0.18–1.39 kg), and a mean total body length increase of 5.5 ± 1.5 cm (range: 0–11 cm) (Table 2 and S2 Table).

**Table 2:** Biometric measurements (Mean ± SE) of recaptured Chinese giant salamanders (n = 8) at the beginning and the end of the study.

	Release	Recapture	Percent Change	Difference (*P*-value)
Body mass (kg)	1.5 ± 0.1	2.0 ± 0.2	32.5	*P* = 0.012
Total body length (cm)	63.4 ± 1.5	68.9 ± 1.7	8.7	*P* = 0.027
Snout-vent length (cm)	39.5 ± 0.6	42.1 ± 1.0	6.6	*P* = 0.027

**Fig 4:**
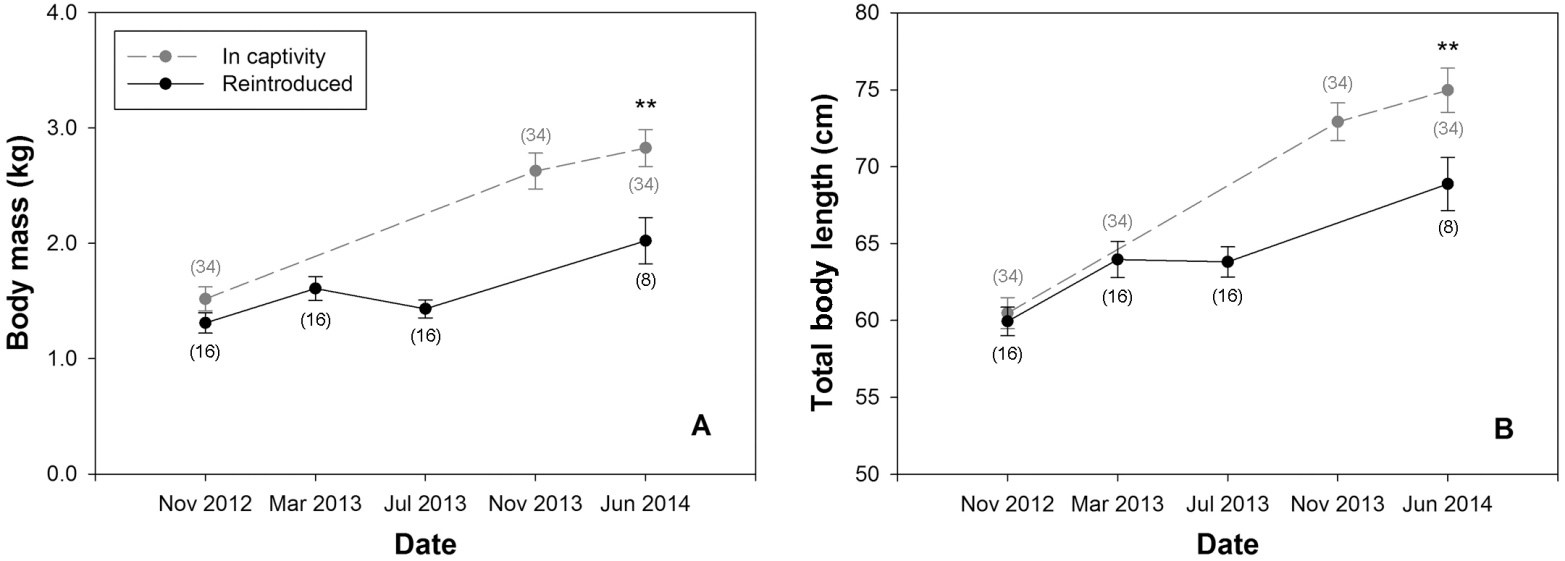
Comparison of body mass and total body length between reintroduced salamanders and their conspecifics in captivity. (A) Body mass comparison. (B) Total body length comparison. The 16 salamanders reintroduced to the Donghe River were from a group of salamanders whose growth was monitored since 2012. They were compared with salamanders that remained in captivity during the entire study period (November 2012–June 2014). Plotted values are means ± 1 SE; ** indicates different values between the two groups at that particular time interval (*P* < 0.01); numbers in parentheses indicate sample size.

The 16 giant salamanders released at the Donghe River were as heavy as their conspecifics reared in captivity before separation (1.31 ± 0.09 vs. 1.52 ± 0.10 kg, Mann-Whitney U = 226.5, *P* = 0.343), and as long (59.9 ± 0.9 vs. 60.5 ± 1.0 cm, Mann-Whitney U = 265, *P* = 0.884). Yet, after surgery and following 11 months in the wild, the eight animals recaptured were lighter than their conspecifics (2.02 ± 0.20 kg vs. 2.83 ± 0.16, Mann-Whitney U = 65, *P* = 0.022), and shorter (68.9 ± 1.7 vs. 75.0 ± 1.4 cm, Mann-Whitney U = 69, *P* = 0.031) (Fig 4).

**Fig 5:**
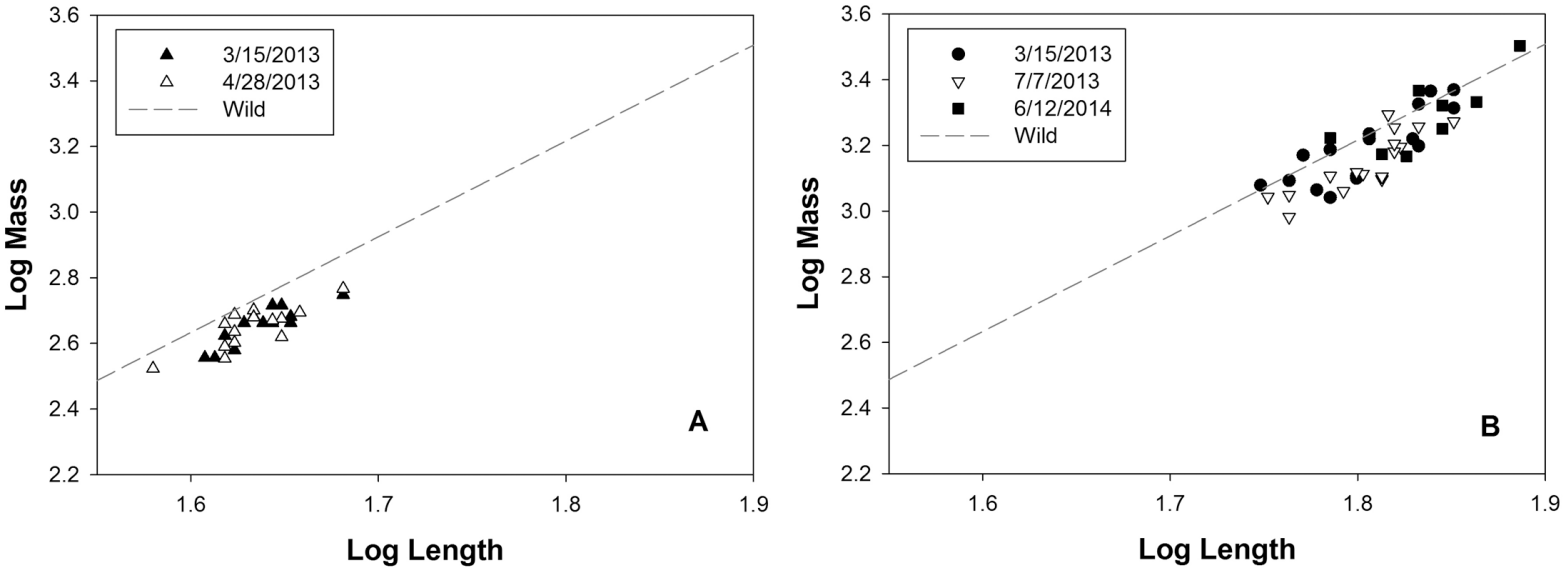
Change in body condition of reintroduced salamanders, compared to wild-caught conspecifics. (A) Salamanders reintroduced at the Heihe River. (B) Salamanders reintroduced at the Donghe River. The regression line: log[Mass] = -2.039+2.920*log[TBL] (R^2^ = 0.98, *P* < 0.001) was constructed using data obtained from previously published papers, indicating body condition of wild-caught giant salamanders [20]. A dot above the regression line indicates that this animal has better body condition than wild salamanders with the same total body length, whereas a dot under the regression line indicates a worse body condition.

Almost all salamanders in the Heihe River group were below the regression line constructed using data from wild animals either at the time of surgery or before release (Fig 5A), indicating they were in poorer condition (had less energy reserve) than wild conspecifics with the same length. The mean residuals were -0.080 ± 0.010 and -0.080 ± 0.011, respectively, with no difference between the two (Mann-Whitney U = 107, *P* = 0.854).

Some of the giant salamanders recaptured at the Donghe River were above the regression line (Fig 5B), indicating that they were in better body condition (had more energy reserve) than wild conspecifics with the same length. However, all giant salamanders had negative mean residuals through the study timeline: from -0.038 ± 0.015 at surgery to -0.083 ± 0.012 at release, and -0.033 ± 0.025 after approximately a year in the wild, although the differences were not statistically significant (χ^2^ = 4.90, *P* = 0.086). Converting to body mass differences, recaptured giant salamanders by the end of the study were only 7% lighter than wild salamanders with the same length.

### Health

Prior to release, two giant salamanders had scars on their bodies, two had wounds on their legs, two had tail tears, and another one had hypertrophy of one digit on its leg. Among the 8 giant salamanders that were recaptured at the Donghe River, one had a tail tear, and another one had a digit on its left hind leg missing compared to pre-release. Both salamanders were different from the ones who had abnormalities before release (S2 Table). No external parasites were found on any of the giant salamanders before and after release. None of the animals recaptured were lethargic and were quite aggressive when handled.

### Prey Species Abundance

We recorded three fish species at the Heihe River in traps, including *Phoxinus lagowskii*, *Paracobitis variegatus*, and *Sarcocheilichthys nigripinnis*, and tadpoles which could not be identified to species. At the Donghe River, we recorded two fish species, *Phoxinus lagowskii*, and *Paracobitis variegatus*, one crab *Sinopotamon* sp., and tadpoles, which could not be identified to species. The average prey mass per trap at the Heihe River was 210.7 ± 34.5 g, which was significantly greater than prey mass of 80.3 ± 21.6 g at the Donghe River (Mann-Whitney U = 58.5, *P* < 0.001).

## Discussion

Here we describe one of the first attempts to reintroduce captive-reared Chinese giant salamanders into the wild and monitor their survival continuously for more than a year through radio telemetry. Our data indicated that more than half of the reintroduced giant salamanders could survive their first year in the wild when animals were given enough time to fully recover from transmitter implant surgery, i.e. the group of salamanders released at the Donghe River. The Donghe group of animals had a comparable annual survival rate (0.70) to that of wild hellbenders (*Cryptobranchus alleganiensis*) (0.81) [23] and reintroduced hellbenders (0.75) [16], suggesting that these animals have a good chance to survive at this river location. Continued monitoring of the salamanders will help reveal their long-term survival, with the help of PIT-tags. These PIT tags were helpful in identifying released individuals after we lost their radio signals, e.g. we successfully caught two giant salamanders from the Donghe group in a later recapture effort in October 2014.

Dehiscing sutures caused 50% of the total 10 confirmed deaths (including the one that died before release). Because there were no reported studies on how long Chinese giant salamanders should be held prior to release following surgery, we modeled our timeline after similar studies on hellbenders. Bodinof et al. [16] suggested releasing hellbenders 14 to 28 days after surgery, and their results showed survival of the released hellbenders decreased as individuals were held longer following surgery. The Heihe group of giant salamanders were held 6 weeks after surgery and the Donghe group were held 16 weeks, much longer than what Bodinof *et al*. [16] suggested. However, the Heihe group of giant salamanders experienced dehiscence both before (n = 2) and after release (n = 4), with a high chance of death (83%) following suture rupture. In contrast, no salamander in the Donghe group had experienced dehiscence. Survival analyses in MARK revealed that regardless of group, survival rate of released salamanders increased as they were held longer from surgery to release (Fig 3), which was opposite to what Bodinof et al. [16] reported. It may be that the younger age of the Heihe group of salamanders, compared to the hellbenders in Bodinof et al. [16] study, had an effect on dehiscence, whereas the Donghe group were similar to the hellbenders in age. Younger animals have thinner skin layers and may have a slower recovery rate than older animals [17]. Thus, younger animals may need more time to recover after surgery, which shortens the valuable post-release monitoring duration. We suggest releasing older giant salamanders for similar studies in the future, while allowing for complete surgical recovery. However, older giant salamanders are more expensive to purchase or to rear in research facilities, due to their larger body sizes. Future reintroduction projects with limited budgets may need to consider the trade-off between fewer large study animals, which may contribute to reproduction sooner, or a greater number of small animals with a shorter monitoring period (due to longer recovery time following surgery) that may have a higher mortality before they are able to reproduce in the wild.

For a short period following surgery, all salamanders in the Donghe group stopped growing or even lost body mass, which eventually resulted in differences of body mass and total body length between these animals and their siblings retained in captivity without surgery (Fig 4). Thus, along with other negative impacts such as dehiscence, surgical implantation of transmitters potentially slowed the growth of the salamanders. There are few studies examining the effects of surgical implantation of transmitters on the growth of salamanders, whereas for fish, e.g., chinook salmon (*Oncorhynchus tshawytscha*) [24] and rainbow trout (*Oncorhynchus mykiss*) [25], no significant effect was found. It appears the effects of surgery on giant salamander growth are opposite to previous reports on fish and this should be considered in similar future telemetry studies. For reintroduced animals, body condition may be more important than body size, as individuals with higher energy reserve may have higher survival rate [26–28]. By the end of our project, the Donghe animals were only 7% lighter than wild giant salamanders with the same length, indicating that they had comparable energy reserve with wild conspecifics to help them survive in the wild. The growth of the Donghe salamanders lent additional support to our assumption that this river can still support these giant salamanders.

Despite the problems related to dehiscence of surgical sites from the earlier released animals and impeded growth as discussed above, implanted VHF radio transmitters usually provide more stable and long-lasting tracking signals compared to external transmitters [29]. For example, Zheng and Wang [15] reported two transmitters dropped off their four monitored giant salamanders within five months, whereas in our case, only the loss of two salamander signals suddenly at the early stage without any floods having occurred were possibly due to transmitter failure or poaching. However, if target animals do not heal thoroughly after surgery, transmitter implantation may largely affect their post-release survival, as revealed in our study and other studies on hellbenders [16]. In contrast, the use of external transmitters had no such pitfalls, and this may contribute to the 100% survival of the four released animals in the study by Zheng and Wang [15]. We would recommend using external transmitters when close monitoring is feasible, such that transmitters lost or damaged could be detected promptly, animals could be recaptured relatively easily and additional funds for purchasing backup transmitters are available. Otherwise, implanted transmitters may be a better choice to obtain useful information when not monitored as frequently or the feasibility of recapturing animals is low.

Floods were only directly involved in the death of one giant salamander that we could determine. However, eight more animals were washed downstream by floods during the rainy seasons, including five animals at the Heihe River, which disappeared eventually from our search radius, and three animals at the Donghe River which remained within 2 km downstream from their original locations. Although, two of them were never recaptured and their status could not be determined, we did recapture one Donghe River salamander and found it had increased in body mass and length since release. We suggest two possible factors for having lost so many animals in floods at the Heihe River compared to the Donghe River. First, the smaller animals at the Heihe River were not fully recovered from surgery and may have been more vulnerable to the damaging nature of being swept up in a flood. Second, the river characteristics themselves may have impacted how well animals could respond to adverse weather conditions. Based on animals that were available for release, we selected the two river sections that are of moderate size and with sufficient boulders for the salamanders to hide beneath [15,16]. However, the Heihe River is slightly wider and the water volume and flow rate fluctuate more than the Donghe River during floods, thus creating a harsher environment for reintroduced salamanders. Survival analysis in MARK supports this hypothesis; the Donghe group had a higher survival rate than the Heihe group. However, as the site model and age model revealed the same results, the effect of site may also be influenced by the animal’s age, or the combination of the two.

Prey and predator are key habitat factors to consider when establishing a reintroduced population [30]. We have surveyed prey species in both rivers, but cannot make a judgment on foraging resources without additional background information on what the habitat was like when wild giant salamanders were abundant in both rivers. As all recaptured giant salamanders at the Donghe River increased in mass and length, and maintained their body condition close to wild conspecifics after almost one year in the wild, we may infer that the Donghe River was able to support these giant salamanders for a short period of time. We were not successful in recapturing any of the giant salamanders that remained at the Heihe River. However, since the traps set at Heihe captured larger amounts of prey than those at Donghe, we may infer that prey was not a limiting factor to the giant salamanders’ survival at the Heihe River. Continued monitoring of these animals may reveal more insight into their habitat requirements. No studies on predators of Chinese giant salamanders have been reported, and few studies on its close relative, the Japanese giant salamanders (*Andrias japonicas*), were available. Local people have suggested Eurasian otters (*Lutra lutra*) as a potential predator, while Zheng and Wang [15] pointed out that other carnivores such as red fox (*Vulpes vulpes*), Siberian weasel (*Mustela sibirica*), and hog badger (*Arctonyx collaris*) may also prey on the giant salamanders. However, none of the nine dead giant salamanders died due to predation, rather they died from dehiscence and floods, or from unknown reasons with their bodies still remaining in the rivers. The two animals, whose radio signals disappeared suddenly without any floods having occurred, were not considered preyed upon, because we did not recover any transmitters alone, nor did we receive radio signals in the nearby forests along the river. Thus, natural predation did not appear to be a main threat to reintroduced giant salamanders, although we cannot eliminate the possibility that salamanders washed downstream from a flood and were lost to the study, did not move into the food chain.

No external parasites were found on recaptured giant salamanders, nor were any confirmed deaths directly associated with physical signs of disease upon necropsy. Ranavirus has been reported in farmed Chinese giant salamanders [31,32]. However, we did not find overt signs of Ranavirus infection (ulcerus lesions, discolored skin or digit loss) in our source population from the farm or in recaptured individuals. Chytrid fungus (*Batrachochytrium dendrobatidis*) is commonly found in amphibians and has caused mass mortalities in several continents [33,34]. To date, this fungus has been found in hellbenders [35] and Japanese giant salamanders [36], yet no chytrid fungus infection has been reported to impact Chinese giant salamanders to our knowledge. Skin swabs are being collected from farmed and recaptured giant salamanders to continually monitor for chytrid fungus or Ranavirus outbreak in case symptoms are observed.

Poaching was not detected during the study, although the two giant salamanders whose signals disappeared within one day without any flood having occurred were suspicious. The market price of the Chinese giant salamander has declined dramatically from $200 USD/kg in 2009 to $20–30 USD/kg in 2014, which is believed to have reduced the number of animals poached from the wild. Public education and media attention can also contribute to the prevention of poaching. Ceremonies occurred at both sites, where staff of local governmental agencies, journalists, and leaders of local communities were invited to join the release. We also hired and trained the leaders of local communities at both sites to monitor the salamanders. These early interventions to poaching may have helped to prevent local people from harvesting reintroduced animals in both rivers, as more information available to local communities regarding little-known and threatened species will result in more positive attitudes toward their protection [37].

A viable, self-sustaining population in the wild represents a successful reintroduction program, and the population must be monitored long enough to determine its status [6,38]. Even though 10 giant salamanders at the Donghe River were alive by the end of our project, we are far from claiming that our project is successful. This group of salamanders would be ~ 7 years old and possibly be sexually mature by the summer of 2015. New recruits due to reproduction could be expected during this breeding season or in the year after, and this would be a further step towards a viable reintroduced population. Moreover, additional giant salamanders with different genetic backgrounds may need to be released at the same site in order to avoid inbreeding within the founder groups, as genetic makeup of the reintroduced population has an important impact on persistence of reintroduced populations [30].

## Supporting Information

S1 Table Status of the 31 salamanders reintroduced into the Heihe and Donghe rivers (2013–2014). (DOCX)

S2 Table Biometric measurements and abnormalities of reintroduced giant salamanders at the beginning and the end of the study (2013–2014). (DOCX)
