# Supplementary material for: Reintroduction and Post-Release Survival of a Living Fossil: The Chinese Giant Salamander
Source: PLoS One. 2016 Jun 3;11(6):e0156715. doi: 10.1371/journal.pone.0156715 (PMC4892505; doi:10.1371/journal.pone.0156715)
Supplement: S1 Table — (DOCX) [file pone.0156715.s001.docx]

**S1 Table.** **Status of the 31 salamanders reintroduced into the Heihe and Donghe rivers (2013 – 2014).**

| Radio frequency | Release date | Release site | Status by the end of the project | Details |
| --- | --- | --- | --- | --- |
| 150.202 | 04/28/2013 | Heihe | Dead | With dehiscence of suture site, recaptured on 04/29/2013; died on 05/05/2013 |
| 150.235 | 04/28/2013 | Heihe | Alive | Re-sighted using underwater camera |
| 150.251 | 05/01/2013 | Heihe | Dead | Crushed during floods, recaptured on 06/14/2013; died on 06/16/2013 |
| 150.273 | 05/01/2013 | Heihe | Undetermined | Washed away by floods, signal could not be found since 10/11/2013 |
| 150.291 | 05/02/2013 | Heihe | Dead | Covered by *Saprolengnia*, Recaptured on 05/05/2013; died on 05/05/2013 |
| 150.351 | 04/28/2013 | Heihe | Dead | With dehiscence of suture site, recaptured on 05/04/2013; died on 05/13/2013 |
| 150.392 | 05/01/2013 | Heihe | Dead | With dehiscence of suture site, recaptured on 05/12/2013; died on 05/14/2013 |
| 150.412 | 11/05/2013 | Heihe | Undetermined | Suture site opened before release; re-sutured and released in November 2013; Signal disappeared on 05/24/2014 during floods |
| 150.431 | 05/01/2013 | Heihe | Undetermined | Washed away by floods, signal could not be found since 10/11/2013 |
| 150.472 | 05/02/2013 | Heihe | Undetermined | Washed away by floods, signal could not be found since 07/22/2013 |
| 150.531 | 04/28/2013 | Heihe | Alive | Re-sighted using underwater camera |
| 150.571 | 04/28/2013 | Heihe | Undetermined | Moved into an underground stream, probably alive although signal could not be detected since 06/18/2013 |
| 150.610 | 05/02/2013 | Heihe | Undetermined | Signal disappeared suddenly on 06/14/2013 without floods having occurred |
| 150.710 | 04/28/2013 | Heihe | Dead | With dehiscence of suture site, recaptured on 05/02/2013; died on 05/12/2013 |
| 150.730 | 05/02/2013 | Heihe | Undetermined | Washed away by floods, signal could not be found since 07/22/2013 |
| 150.101 | 07/12/2013 | Donghe | Alive | Recaptured |
| 150.211 | 07/12/2013 | Donghe | Alive | Recaptured |
| 150.312 | 07/12/2013 | Donghe | Dead | Died on 12/31/2013 with undetermined cause |
| 150.332 | 07/12/2013 | Donghe | Alive | Recaptured |
| 150.371 | 07/12/2013 | Donghe | Alive | Recaptured |
| 150.450 | 07/12/2013 | Donghe | Alive | Re-sighted using underwater camera |
| 150.490 | 07/12/2013 | Donghe | Alive | Moved downstream during floods; recaptured alive by the end of the project |
| 150.511 | 07/12/2013 | Donghe | Alive | Re-sighted using underwater camera |
| 150.550 | 07/12/2013 | Donghe | Dead | Died on 10/15/2013 with undetermined cause |
| 150.591 | 07/12/2013 | Donghe | Undetermined | Moved downstream during floods; signal disappeared on 4/23/2014 during floods |
| 150.630 | 07/12/2013 | Donghe | Undetermined | Moved downstream during floods; neither recaptured nor re-sighted |
| 150.651 | 07/12/2013 | Donghe | Undetermined | Signal disappeared suddenly on 09/27/2013 without floods having occurred |
| 150.671 | 07/12/2013 | Donghe | Alive | Recaptured |
| 150.751 | 07/12/2013 | Donghe | Alive | Recaptured |
| 150.770 | 07/12/2013 | Donghe | Dead | Died on 10/14/2013 with undetermined cause |
| 150.790 | 07/12/2013 | Donghe | Alive | Recaptured |
