# Supplementary material for: Reintroduction and Post-Release Survival of a Living Fossil: The Chinese Giant Salamander
Source: PLoS One. 2016 Jun 3;11(6):e0156715. doi: 10.1371/journal.pone.0156715 (PMC4892505; doi:10.1371/journal.pone.0156715)
Supplement: S2 Table — (DOCX) [file pone.0156715.s002.docx]

**S2 Table.** **Biometric measurements** **and abnormalities of reintroduced giant salamanders at the beginning and the end of the project (2013 – 2014).**

| Radio frequency | Release site | Body condition before release | | | ^a^Abnormalities before release | Body condition by the end of the project | | | Abnormalities by the end of the project |
| --- | --- | --- | --- | --- | --- | --- | --- | --- | --- |
|  |  | Length (cm) | SVL (cm) | Mass (g) |  | Length (cm) | SVL (cm) | Mass (g) |  |
| 150.412 | Heihe | 42 | 27 | 400 | × |  |  |  |  |
| 150.273 | Heihe | 41.5 | 26.5 | 389.6 | × |  |  |  |  |
| 150.710 | Heihe | 44.5 | 28 | 416.8 | Hypertrophy of one digit on the left front leg |  |  |  |  |
| 150.531 | Heihe | 44.5 | 27.5 | 473.2 | × |  |  |  |  |
| 150.610 | Heihe | 48 | 29 | 584.8 | × |  |  |  |  |
| 150.251 | Heihe | 55.5 | 43 |  | × |  |  |  |  |
| 150.472 | Heihe | 41.5 | 27 | 456.1 | × |  |  |  |  |
| 150.351 | Heihe | 45.5 | 27 | 494.9 | × |  |  |  |  |
| 150.235 | Heihe | 42 | 25.5 | 487.4 | × |  |  |  |  |
| 150.202 | Heihe | 44 | 28 | 468.5 | × |  |  |  |  |
| 150.730 | Heihe | 42 | 26 | 432 | × |  |  |  |  |
| 150.291 | Heihe | 38 | 24.7 | 333.8 | Scar on upper belly |  |  |  |  |
| 150.571 | Heihe | 41.5 | 26 | 358.4 | × |  |  |  |  |
| 150.392 | Heihe | 43 | 27 | 502.9 | × |  |  |  |  |
| 150.431 | Heihe | 43 | 27 | 478.4 | × |  |  |  |  |
| 150.691 |  |  |  |  |  |  |  |  |  |
| 150.490 | Donghe | 66 | 40.5 | 1800 | × | 77 | 44 | 3190 | × |
| 150.450 | Donghe | 63.5 | 39 | 1300 | × |  |  |  |  |
| 150.770 | Donghe | 71 | 42.5 | 1875 | × |  |  |  |  |
| 150.371 | Donghe | 58 | 38 | 1120 | Tail tears | 67 | 42 | 1470 | × |
| 150.651 | Donghe | 65 | 38 | 1250 | × |  |  |  |  |
| 150.312 | Donghe | 63 | 38 | 1315 | Tail tears |  |  |  |  |
| 150.332 | Donghe | 68 | 42 | 1810 | Right front leg wounded, one digit on the left hint leg wounded | 68 | 42 | 2330 | × |
| 150.630 | Donghe | 66.5 | 39 | 1570 | × |  |  |  |  |
| 150.511 | Donghe | 62 | 41 | 1150 | Left hint leg wounded |  |  |  |  |
| 150.591 | Donghe | 65 | 39 | 1275 | × |  |  |  |  |
| 150.211 | Donghe | 66 | 39 | 1520 | × | 70 | 41 | 1780 | One tail tear |
| 150.550 | Donghe | 58 | 40 | 960 | × |  |  |  |  |
| 150.101 | Donghe | 56.5 | 36.5 | 1105 | × | 65 | 40 | 1490 | One digit on the left hint leg missing |
| 150.790 | Donghe | 65.5 | 40 | 1970 | Scar on chin | 73 | 47 | 2150 | × |
| 150.751 | Donghe | 61 | 39 | 1280 | × | 61 | 38 | 1670 | × |
| 150.671 | Donghe | 66 | 41 | 1605 | × | 70 | 43 | 2100 | × |

^a^Physical abnormalities includes: tail notches, tail tears, digit missing, supernumerary, fused, reduced, abrasion, and scar. “×” indicates no abnormality was observed.
